# Microbial dark matter sequences verification in amplicon sequencing and environmental metagenomics data

**DOI:** 10.3389/fmicb.2023.1247119

**Published:** 2023-11-02

**Authors:** Hana Barak, Naomi Fuchs, Michal Liddor-Naim, Irit Nir, Alex Sivan, Ariel Kushmaro

**Affiliations:** ^1^Department of Civil and Environmental Engineering, Ben-Gurion University of the Negev, Beer-Sheva, Israel; ^2^Avram and Stella Goldstein-Goren Department of Biotechnology Engineering, Ben-Gurion University of the Negev, Beer-Sheva, Israel; ^3^The Ilse Katz Center for Nanoscale Science and Technology, Ben-Gurion University of the Negev, Beer-Sheva, Israel; ^4^School of Sustainability and Climate Change, Ben-Gurion University of the Negev, Beer-Sheva, Israel

**Keywords:** metagenomics, microbial dark matter, microbial community, amplicon sequencing, bacteria

## Abstract

Although microorganisms constitute the most diverse and abundant life form on Earth, in many environments, the vast majority of them remain uncultured. As it is based on information gleaned mainly from cultivated microorganisms, our current body of knowledge regarding microbial life is partial and does not reflect actual microbial diversity. That diversity is hidden in the uncultured microbial majority, termed by microbiologists as “microbial dark matter” (MDM), a term borrowed from astrophysics. Metagenomic sequencing analysis techniques (both 16S rRNA gene and shotgun sequencing) compare gene sequences to reference databases, each of which represents only a small fraction of the existing microorganisms. Unaligned sequences lead to groups of “unknown microorganisms” that are usually ignored and rarefied from diversity analysis. To address this knowledge gap, we analyzed the 16S rRNA gene sequences of microbial communities from four different environments—a living organism, a desert environment, a natural aquatic environment, and a membrane bioreactor for wastewater treatment. From those datasets, we chose representative sequences of potentially unknown bacteria for additional examination as “microbial dark matter sequences” (MDMS). Sequence existence was validated by specific amplification and re-sequencing. These sequences were screened against databases and aligned to the Genome Taxonomy Database to build a comprehensive phylogenetic tree for additional sequence classification, revealing potentially new candidate phyla and other lineages. These putative MDMS were also screened against metagenome-assembled genomes from the explored environments for additional validation and for taxonomic and metabolic characterizations. This study shows the immense importance of MDMS in environmental metataxonomic analyses of 16S rRNA gene sequences and provides a simple and readily available methodology for the examination of MDM hidden behind amplicon sequencing results.

## 1. Introduction

The most diverse and abundant life form on planet Earth, microorganisms play a fundamental role in the planet’s ecosystem health by cycling nutrients, degrading environmental pollutants, facilitating primary production, and providing essential nutrients and chemicals such as oxygen and different vitamins that humans and animals cannot produce themselves (Morowitz et al., 2011; Rinke et al., 2013; Solden et al., 2016). The conventional methods of studying these microorganisms and to elucidate their capabilities have, in the past, relied on already well-developed, classical laboratory techniques, in particular the use of cultivation methods. Nonetheless, in many environments only limited numbers of microorganisms have been cultivated to date (Solden et al., 2016; Zamkovaya et al., 2021). The famous “great plate count anomaly” is one of the earliest depictions of the gap between the actual number of bacteria present in a given sample and the much smaller number that can be effectively cultivated (Staley and Konopka, 1985). The extent of microorganism diversity was further elucidated by analyzing microbial ribosomal RNA (rRNA) gene sequences directly collected from environmental samples (Baker and Dick, 2013). During the last few decades, the 16S rRNA gene has emerged as the most sequenced taxonomic marker (Tringe and Hugenholtz, 2008), forming a cornerstone for systematic classification that is also exploited as a genetic marker to infer the phylogenetic relationships among prokaryotes.

The use of metabarcoding based on short variable region sequencing of the 16S rRNA gene has revolutionized microbial ecology, allowing for the rapid and high-throughput identification of complex microbial communities (Santos et al., 2020). However, due to the short amplicon lengths used in this analysis, this approach has limitations in the extent to which it can accurately affiliate microbial taxa to species or even genus levels, a resolution that is insufficient for differentiating closely related taxa. In addition, this method is prone to PCR amplification biases, sequencing errors, and variations in the copy number of the 16S rRNA gene across different taxa. To address these limitations, recent strategies have been developed that enable nearly full-length sequencing of the 16S rRNA gene, improving the accuracy of microbial identification and facilitating the discovery of novel taxa. Included among these approaches are long-read sequencing technologies such as PacBio and Oxford Nanopore and hybrid sequencing approaches that combine short-read and long-read sequencing technologies. These methods provide higher resolution and more accurate taxonomic classification, thereby increasing the reliability of microbial identification in various research fields. Nevertheless, Illumina short variable region sequencing is still the standard sequencing technology and the most frequently used method in microbial ecology studies. The importance of 16S rRNA gene sequences to the field notwithstanding, an exclusive reliance on this analytical method may fail to provide complete information about bacterial classification. According to Yarza et al. (2014), a sequence identity of 94.5% or lower for two 16S rRNA genes provides strong evidence that they belong to distinct genera, while lower sequence identities of 86.5% correspond to families, 82% to orders, 78.5% to classes and 75% to phyla. Analyses of the 16S rRNA gene from environmental samples revealed that fewer than half of the known microbial phyla are represented by at least one cultivated representative. Moreover, among all microbial isolates, more than 88% belong to only four bacterial phyla (from among the more than 1,500 estimated phyla): Proteobacteria, Firmicutes, Actinobacteria and Bacteroidetes (Rinke et al., 2013; Solden et al., 2016). To date, the phyla that contain only uncultured representatives, identified via the phylogenetic analysis of rRNA genes recovered from environmental samples, have been referred to as candidate phyla. Lacking the support of bacterial culture results, rRNA based sequence analysis alone is unable to classify the majority of the microbial population. Microbiologists have therefore compared the problem of this “uncultured microbial majority” to that of “dark matter” in astrophysics, adopting similar terms such as “microbial dark matter” (MDM) to describe the uncultivated microbes (Hedlund et al., 2014; Jiao et al., 2021). Among the MDM, one prominent group of candidate phyla radiation (CPR) is known by the super-phylum name Patescibacteria (Harris et al., 2004; Nakai, 2020).

Genomic analyses of CPR representatives showed that metabolic limitations have prevented our ability to cultivate these organisms, which are typically smaller than cultivated bacteria (∼0.2 microns) (Vigneron et al., 2020) and with shorter genomes (∼1 Mbp). Moreover, they often have unusual ribosome compositions that contain self-splicing introns and proteins encoded within their rRNA genes, a feature rarely reported in bacteria (Brown et al., 2015). Many are thought to be unable to produce their own nucleotides and are believed to possess minimal amino acid contents and limited cofactor biosynthetic capacity. Indeed, analyses of their genomes showed that they lack CRISPR (Tian et al., 2020) and the components necessary to synthesize membrane lipids (Castelle and Banfield, 2018). Nevertheless, their genomes have been recovered from diverse environments ranging from the human microbiome to drinking water to marine and deep subsurface sediments and soil (Méheust et al., 2019). A recent phylogenetic study found that protein family presence/absence patterns cluster the Patescibacteria super-phyla together and separate from all other bacteria and archaea.

Debate over the extent of the MDM diversity has led to estimates that it could account for as much as 25–50% of all bacterial diversity (Hug et al., 2016; Parks et al., 2017; Schulz et al., 2017). The inability to definitively determine its contribution to diversity may be because some of its groups are not detected in 16S rRNA gene taxonomic and diversity surveys due to primer mismatch and/or to the presence of introns within their 16S rRNA gene that may interfere with polymerase chain reaction (PCR) amplification (Castelle and Banfield, 2018). There is accumulating evidence that these uncultivated microorganisms account for a larger portion of the Earth’s biomass and biodiversity than was previously thought, reflecting the profound bias of the current body of knowledge about microbial life.

In metagenomic sequencing analysis (both 16S rRNA gene and shotgun sequencing), sequences are compared to reference databases that contain only a small part of the existing microorganisms. This results in uncovering of groups of yet unclassified microorganisms. Despite the increasing awareness of their immense importance, these unclassified amplicon sequences, designated by us as “microbial dark matter sequences” (MDMS), are usually ignored or discarded during typical microbial community profiling studies.

The aim of this study, therefore, was to provide additional support for the immense importance of MDMS in environmental metataxonomic analyses using the 16S rRNA gene. To that end, we analyzed 16S rRNA gene sequences collected from four different, highly diverse environments—a living organism, rocks from a desert environment, natural aquatic environments and a membrane bioreactor for wastewater treatment. Our ongoing studies of the varied microbiomes of these environments availed us of the necessary samples from each environment. Of the sequences collected, 163 16S rRNA representative gene sequences, obtained from amplicon sequencing, were chosen for additional examination as potential MDMS. These sequences were screened against various databases and aligned to the GTDB (Genome Taxonomy Database) to build a comprehensive phylogenetic tree for additional sequence classifications. The putative MDMS were screened against metagenome-assembled genomes from the explored environments for additional validation and for taxonomic and metabolic capacity characterization. Using a relatively simple, currently available methodology, this study sheds additional light on MDMS that will improve our conceptualization of the bacterial diversity in any environment.

## 2. Materials and methods

### 2.1. Total genomic DNA extraction

For the purposes of this study, we used total gDNA obtained from four vastly different environments:

- A membrane bioreactor (MBR) used to treat chemical industry wastewater; system description and DNA extractions described in Barak et al. (2020).
- Larvae of the beetle *Capnodis tenebrionis* (CT); experiment described in Barak et al. (2019).
- Surfaces of Negev desert rocks (NDR)—12 rock samples from two petroglyph sites in the Negev desert of Israel from the Ramat Matred and Har Michya sites; experiment and DNA extraction procedure described in Irit et al. (2019) and Nir et al. (2019).
- Confined and unconfined aquifers—five biomass samples scraped from different coupons made of glass, steel and stainless steel that had been deployed in water wells in the Arava Valley.
- In addition, 20 biomass samples were obtained By sterile filtering 50 L of water from The wells In The Arava Valley using The Stericup-GP sterile vacuum filtration system containing a polyethersulfone membrane with a pore size of 0.22 μm (Merck, Gillingham, United Kingdom). Extraction of total genomic DNA from The biomass samples Was carried Out using The MoBio PowerWater isolation Kit (MoBio laboratories Inc. Carlsbad, CA, United States) and The DNeasy PowerSoil Kit (Qiagen, United States).

### 2.2. Next generation amplicon sequencing

The total genomic DNA that was extracted from the samples was submitted to the DNA Services facility (DNAS) of the Research Resources Center at the University of Illinois Chicago (UIC) for gene sequencing of the bacterial small subunit (16S) of ribosomal RNA (rRNA) using the Illumina MiSeq platform with a sequencing length of 300 bps. Prior to sequencing, two PCR amplification steps were performed. During the first PCR reaction, fragments of the V3–V4 (environments 1–3) and V1–V3 (aquifers) regions of the 16S rRNA gene were amplified using universal primers (341F/806R and 27F/534R, respectively) (Jumpstart Consortium Human Microbiome Project Data Generation Working Group, 2012; Hugerth et al., 2014; Elovitz et al., 2019) to which were attached the 5′ linker sequences CS1 and CS2 (known as common sequence 1 and 2). The second PCR reaction was done to prepare the library as described by Green et al. (2015).

### 2.3. Metataxonomic data analysis

Raw reads were merged using the PEAR software package (v0.9.10) (Zhang et al., 2014), with a quality score threshold of 25 for trimming and a base PHRED quality score of 33. Sequence data were screened to remove low-quality sequences and potentially chimeric sequences with the Mothur software package (v1.36.1) (Schloss et al., 2009). Sequences that contained more than eight bases homopolymers or any ambiguous bases were removed, and a length cutoff of 250 bp was used. The resultant sequences file was screened against the phix 174 genome (ID—MN385565) using BLASTN (Chen et al., 2015) to remove sequencing/processing artifacts. The quality-controlled sequences were then processed with the Qiime software package (v1.9.1) (Caporaso et al., 2010). Briefly, sequence data were clustered into operational taxonomic units (OTU) at 97% similarity. Representative sequences from each OTU were extracted and classified using the “assign_taxonomy.py” script with the UCLUST assignment method, utilizing the SILVA database (Quast et al., 2012).
Representative sequences were also aligned using the “align_seqs.py” script with percent identity thresholds of 75 and 90% to the Silva alignment reference file (Quast et al., 2012). The aligned sequences were filtered using the silva_lanemask_mothur file and then used to produce a phylogenetic tree. Four biological observation matrices (BIOM) (McDonald et al., 2012) were generated at taxonomic levels from phylum to genus using the “make_OTU_table.py” script. Sequences that failed to align with the Silva DB for the above-mentioned thresholds were not included in the BIOM tables. An additional BIOM table was also generated in which no alignment-based sequence filter was applied. The “filter_otus_from_otu_table.py” script ensured that only OTUs with minimum total observation counts of 50 reads were retained. All data analysis was done using the Silva database (v.138) as a reference. BIOM tables were converted from read counts to relative abundances and the relative abundances of the unassigned OTUs from each dataset were plotted to present the differences between 75 and 90% alignment thresholds.
Beta diversity (pairwise sample dissimilarity) was calculated using Bray-Curtis, and a 2D nMDS plot was generated using R.
The OTU table (based on all representative sequences, without eliminating alignment failures) was converted from read numbers to relative abundance values, and OTUs that were not assigned to any known lineage (not even at the phylum level) and that had relative abundance summaries higher than 0.5% were chosen for further observation as putative MDMS (Supplementary Table S6 presents a summarized overview of taxa at the phylum level, derived from the biome table).

### 2.4. Taxonomic analysis of putative MDMS

For a more comprehensive taxonomic classification, the 163 putative MDMS were compared to four different databases using BLASTN (Altschul et al., 1990): the Silva database (v.138) (Quast et al., 2012), EzBioCloud’s 16S database (updated in May 2018) (Yoon et al., 2017), the GTDB (r89) (Parks et al., 2018) and the nucleotide collection database (nt) of the NCBI last accessed in February 2020 (NCBI Resource Coordinators, 2013). Manual observation of the similarity percentage and query cover of the obtained hits for each putative MDMS provide a more accurate taxonomic classification based on similarity percentage as described in Yarza et al. (2014).

### 2.5. Phylogenetic analysis of putative MDMS

To generate a phylogenetic tree that integrates our putative MDMS with the known bacteria, we used the SSU rRNA sequences with lengths of 600–2,000 bases from the GTDB repository (bac120 ssu r89). First, the GTDB SSU rRNA sequences were aligned using the SSU-ALIGN v.0.1 software (Nawrocki, 2009). The aligned sequences were then masked based on posterior probability (PP) annotation at the default value of 0.95 for aligned residues and as a value of 0.70 for the gap threshold based on the frequency of gap characters in each column. Numerous candidates of the CPR super-phylum known to encode insertions were clustered in several locations of these MDMS 16S rRNA genes. The SSU-ALIGN algorithm that was used in the secondary structure-and function-based multiple sequence alignment (MSA) analysis only included parts of the gene that lacked the insertions.

The putative MDMS were added to the GTDB MSA using the MAFFT v7.464 software (with the Addfragments option) (Katoh and Standley, 2013). The full phylogenetic tree was generated based on the merged alignment using FastTree_v2.1.10 (Price et al., 2010). Visualization was carried out using the Interactive Tree of Life (iTOL) online interface (Letunic and Bork, 2016).

### 2.6. MDMS existence validation

Specific primers were designed for about 30 MDMS using Primer-BLAST (Ye et al., 2012). Primers suggested by Primer-BLAST were examined through the Amplifx software for GC content, self-dimer, Tm and annealing to the target sequence. Primers were synthesized by SIGMA-ALDRICH Co., LLC (Rehovot, Israel). The primers were attached to the 5′ linker sequences CS1 and CS2 and the samples originated each MDMS of interest were sent for sequencing using Illumina MiSeq platform by the DNA Services facility (DNAS) of the Research Resources Center at the University of Illinois Chicago (UIC). The obtained sequencing data was analyzed as described in the “Metataxonomic data analysis” section previously. If the amplification was not specific, it was ignored. If it did provide specific OTU, the representative OTU sequence was compared to the original MDMS sequence using blast. Only sequences with high levels of similarity (>95%) and 100% query cover are shown.

Furthermore, targeted chimera check was conducted for all MDMS, utilizing the DECIPHER web tool (v2.27.2) (Firth et al., 2009).

### 2.7. Metagenomic analysis, putative MDMS screening, and genome characterization

Genomic DNA from 17 representative samples from the two environments with abundances of MDMS (NDR and aquifers) were sequenced by the Illumina NextSeq500 platform in the DNA Services (DNAS) Facility of the Research Resources Center at the University of Illinois at Chicago (UIC).

Metagenomic data were processed by the metaWRAP pipeline v1.2.1. Raw reads were subjected to quality control (QC) using TrimGalore v0.5.0 (Krueger, 2012) and low-quality reads were removed. The QC-passed sequences were assembled using MetaSPAdes v3.13.0 (Nurk et al., 2017) (or MEGAHIT v1.1.3 (Li et al., 2015) in Ramat-Matred samples due to memory limitation). The assemblies and the QC-passed sequences were used for metagenomic binning using three different algorithms: MaxBin v2.2.6 (Kang et al., 2015), metaBAT v2.12.1 (Kang et al., 2015), and CONCOCT v1.0.0 (Alneberg et al., 2013). The resulting three bin sets were consolidated to obtain a single, strong bin set with a minimum completion of 50% and a maximum contamination of 10%. The consolidated bin set was reassembled using both “strict” and “permissive” algorithms, and once the reassembled bin had been improved, it replaced the original bin.

The chosen 163 putative MDMS were screened against both the assembly results and the final bins using BLASTN. The results were examined manually based on the percent similarity (>96%) and cover and on the overlap locations.

The consolidated matched MDMS bins were functionally annotated using Prokka v1.13 (Seemann, 2014) with metaWRAP’s Annotate_bins module. Additional metabolic and biogeochemical functional trait profiling was carried out using the METABOLIC profiler software (Zhou et al., 2019) with METABOLIC-C.pl. version 4.0.

See Supplementary Figure S2 for an outline of the methodology pipeline.

## 3. Results and discussion

Microbial community analysis based on 16S rRNA gene amplicon sequencing is a widespread and important technique in microbiological research (Prodan et al., 2020) that allows researchers to characterize the environment and to determine which microorganisms, both cultured and uncultured, are present in an environmental sample. General analyses of the 16S rRNA gene sequences should compare them to relevant databases. Based mainly on laboratory-cultured bacteria, however, these databases (and indeed, most of our knowledge of microorganisms) are relatively limited in scope, thus rendering the resulting notion of the tree of life unable to present a comprehensive picture of the microbial world. Shedding light on the “dark matter” inhabiting the tree of life may therefore improve our understanding of explored environments and contribute to reshaping the microbial world’s taxonomy.

Today’s whole genome shotgun sequencing studies, especially those focused on single-cell sequencing, constitute the leading methods used to explore uncultured microorganisms and expand our knowledge of the microbial world (Jiao et al., 2021; Wiegand et al., 2021). Indeed, this technique has illuminated the understudied “microbial dark matter” (MDM), thereby helping to fill the gaps in the growing tree of life and eventually explain those microorganisms’ roles in the environment. To date, however, phylogenetic studies rely mostly on 16S rRNA sequences and metagenomic shotgun sequencing.

The objective of this study is to fortify our ability to discover the hidden potential of the “microbial dark matter” by using 16S rRNA amplicon sequencing. To achieve this, we performed bioinformatic analyses of 16S rRNA gene sequences obtained from four very different environments representing diverse conditions: (1) A contaminated industrial environment, i.e., a membrane bioreactor used to treat chemical industry wastewater; (2) *Capnodis tenebrionis* as a living habitat; and two natural desert environments, (3) desert rocks with petroglyphs, and (4) water wells (confined and unconfined aquifers) in the Arava Valley. These environments, varied habitats that have yet to be rigorously explored, demonstrate their potential as sources for the discovery of new, unculturable bacteria.

**Figure 1:**
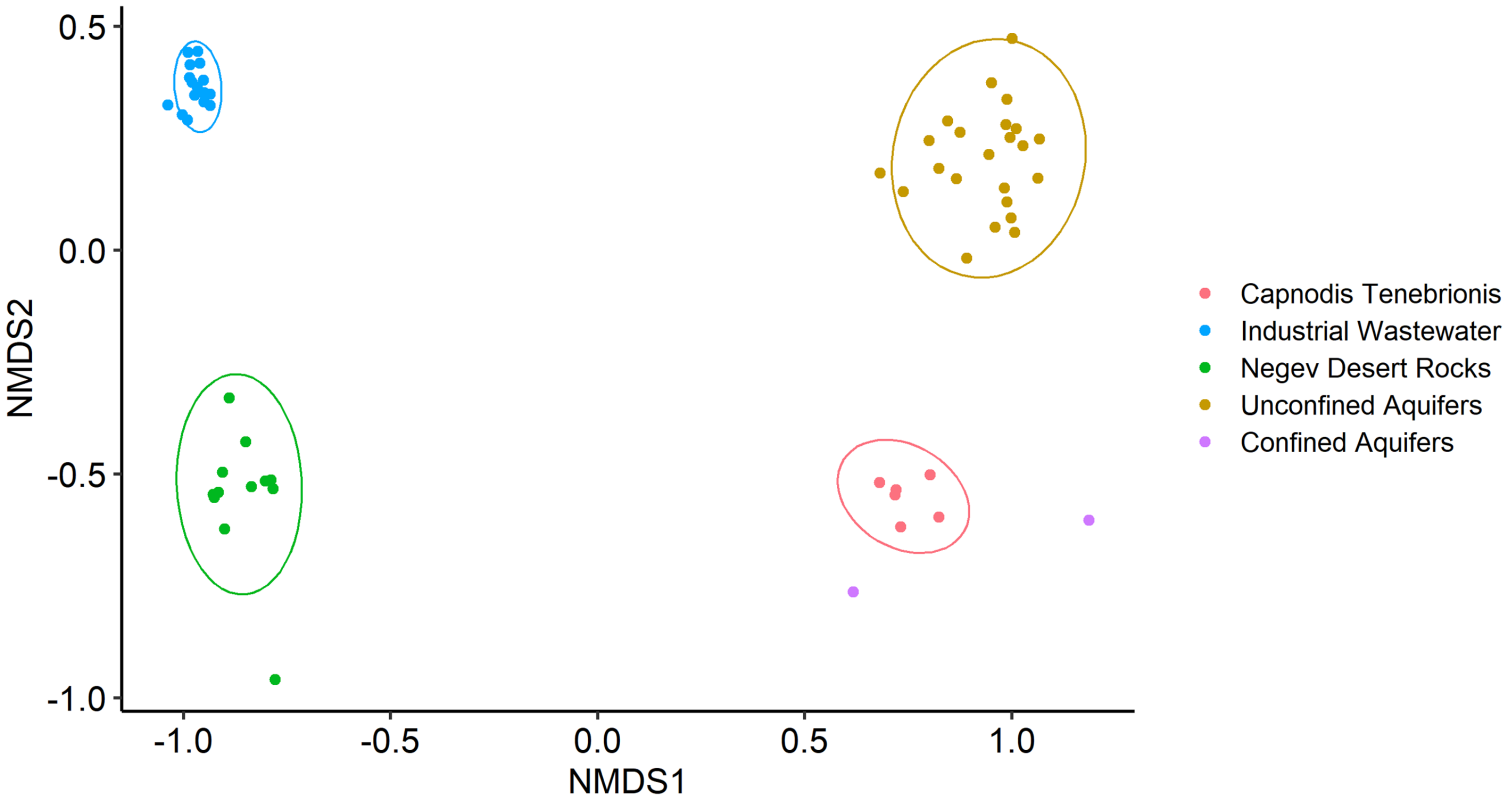
Non-metric multidimensional scaling (nMDS) based on Bray-Curtis, with normal data ellipses (stress level: 0.09).

As expected, non-metric multidimensional scaling (nMDS) analysis (Figure 1) showed high variance between the datasets (confined and unconfined aquifers treated as two separate groups). Anosim and Permanova tests supported this result with a *p*-value of 0.0001 and test statistics of 0.998 and 11.394, respectively.

**Figure 2:**
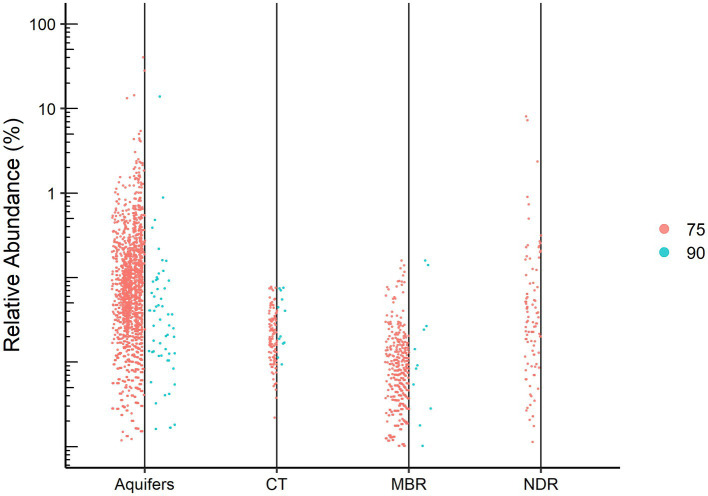
Dots represent the relative abundance of each unassigned OTU for the studied environments: Aquifers (confined and unconfined), CT, Capnodis Tenebrionis; MBR, industrial wastewater (membrane bioreactor); NDR, Negev desert rocks. The identity thresholds are 75% (left) and 90% (right).

Using a set of bioinformatic filters, we generated a total of 5,174,233 high-quality reads obtained from 61 samples. These reads originated from an initial dataset comprising approximately 14 million raw reads. Among the 5,558 representative OTUs with a minimum of 50 repeated observations, 529 OTUs (~9.5%) were not assigned to any known lineage. We found a major difference in the number of unassigned OTUs when data were rarified based on 75 and 90% identity thresholds for alignment (Figure 2), a finding which may indicate that the “dark” part of the microbial environment is located in the gap between the 75 and 90% similarity thresholds. These cutoffs (75 and 90%) were chosen based on the recommended minimum percent similarities to include a sequence in an alignment and to consider a database match a hit, respectively.^1^ Interestingly, natural aquifer water and desert rocks contained higher number of unassigned OTUs in both relative abundance and absolute numbers compared to the engineered environment of the wastewater treatment system. Indeed, according to previous works, unclassified sequences are commonly found in less studied natural environments such as natural water habitats (Keshri et al., 2015; Panda et al., 2017) and semiarid endoliths (Hug et al., 2016). Since aquifer samples were sequenced for the variable regions V1-V3 and all other samples were sequenced for V3–V4, it could also explain part of the differences in the portion of unclassified sequences between the different environments.

The relative abundances of the unassigned OTUs ranged from minor to as high as 40% of the reads obtained from a confined aquifer sample. Indeed, our results together with those of recent works (Zamkovaya et al., 2021) demonstrate that “microbial dark matter” are key ecological players within their respective communities. While Lynch et al. (2012) emphasize the importance of novel phylogenetic diversity in what has been dubbed the “rare biosphere,” wherein they examine low relative abundance sequences, the present study focuses on the highly abundant but uncharacterized sequences. Rare biosphere sequences are liable to be missed by metagenomic sequencing due to the lack of a PCR amplification step (Pascoal et al., 2021).

**Figure 3:**
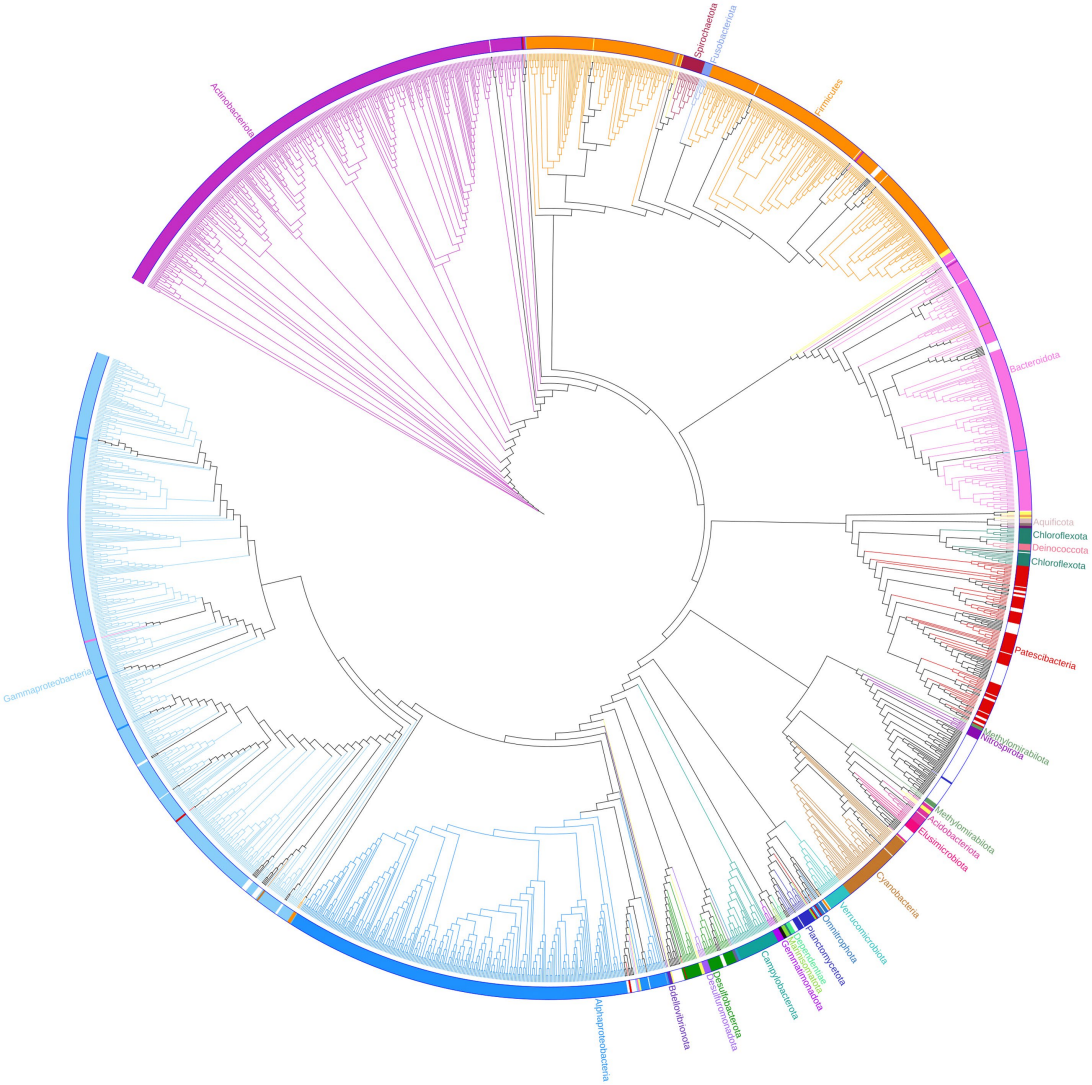
Phylogenetic tree. One hundred and sixty three representatives of the unassigned group are marked with black dots and integrated within the bacteria sequences of the GTDB (bac120_ssu_r89.fna). Branches, strips, and labels are uniquely colored according to phyla.

**Figure 4:**
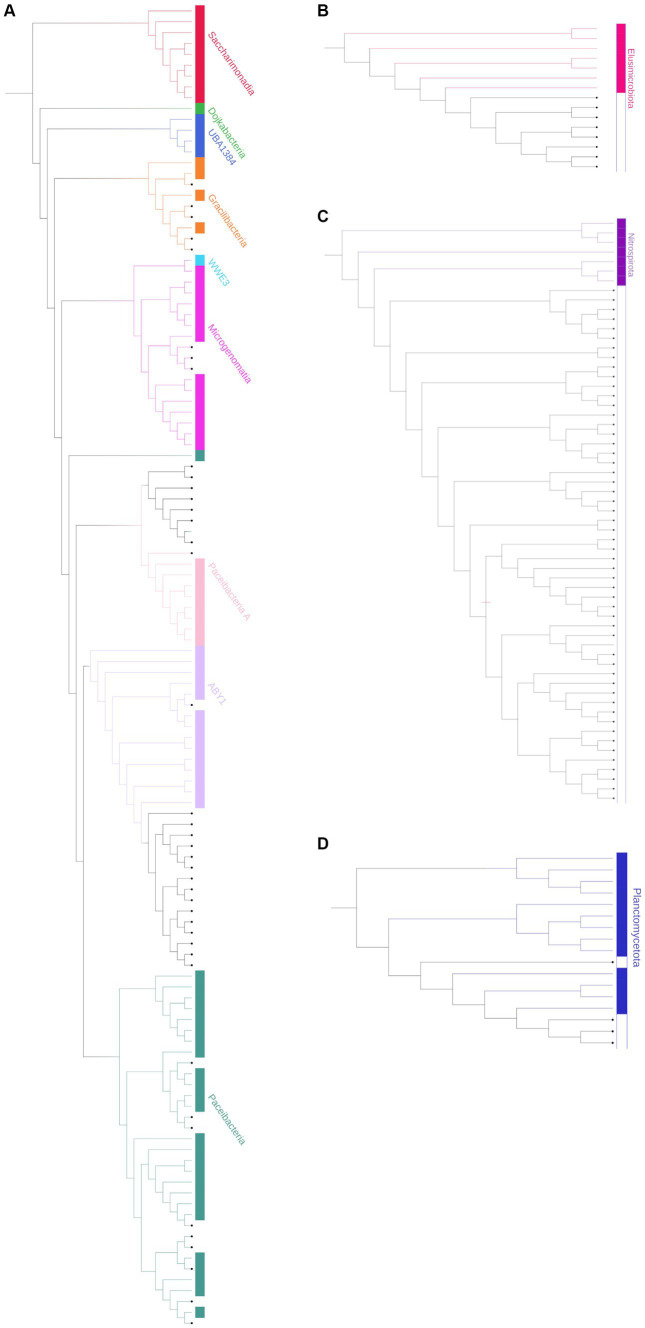
Pruned phylogenetic trees of the **(A)** Patescibacteria super-phylum [Candidate phyla radiation (CPR)], combines 40 representative unassigned OTUs (bold); **(B)** Elusimicrobiota; **(C)** Nitrospirota; and **(D)** Planctomycetota. The tree is pruned from the phylogenetic tree in Figure 3. Branches, strips, and labels are uniquely colored according to phyla.

Based on their relative abundances, 163 of the unassigned sequences were chosen to represent putative MDMS, and these were screened against four different updated databases: Silva, EZ, NCBI, and GTDB. The best match for each MDMS after manual observation is presented in Supplementary Table S1. To enable assumptions about their taxonomic attributions, the putative MDMS were also aligned to the GTDB to build a phylogenetic tree (Figure 3) that was pruned into four smaller trees (Figure 4) to facilitate a more comprehensive perspective of MDMS distribution across the tree of life. A substantial number of the MDMS (40 out of 163) were found to be part of the Patescibacteria super-phylum (Figure 4A). Indeed, it is reasonable that a relatively large portion of the MDMS belongs to the Patescibacteria super-phylum, since they are largely uncultured and therefore understudied. Interestingly, it is still not known whether the distinct phylogenetic position of Patescibacteria in the tree of life is due to rapid evolution by genome reduction or to its early evolutionary split from the non-Patescibacteria (Méheust et al., 2019; Wiegand et al., 2021).

Eight MDMS were related to the Elusimicrobiota and four were related to the Planctomycetota phylum. A group of 53 MDMS, all obtained from aquifer samples, was situated near the Nitrospirota phylum. A tree of putative MDMS from aquifers (Supplementary Figure S1A) suggests that the members of this group do not necessarily belong together. Comparisons of their BLAST results with the GTDB also yielded similarities of 75–85% to different phyla such as Bacteroidota, Methylomirabilota, Desulfuromonadota, Actinobacteriota, Planctomycetota, etc. Nitrospirota have been shown to consistently coexist with Patescibacteria, after which they are the most common phylum in the groundwater population (Herrmann et al., 2019; Yan et al., 2021). Nevertheless, it seems that in our case, not all of the 53 MDMS are part of the Nitrospirota phylum, which may be due to their misclassification.

In the general phylogenetic tree (Figure 3), MDMS were also integrated within different phyla, including the Gammaproteobacteria, Firmicutes, Bacteroidota, Cyanobacteria, etc. We also validated the existence of the putative 16S rRNA MDMS by specific PCR amplification and Mi-Seq Illumina re-sequencing using specific self-designed primers for a few representative MDMS (Table 1). Comparisons of the re-sequenced fragments to the original putative MDMS yielded similarity percentages of 95.91–100%, indicating appropriate primer design and the existence of these sequences in our sequencing data. In the present work, each MDMS is a representative sequence of a group of similar sequences (97% similarity) that constitute an OTU. Previous works found that distinct taxa may be found within a single OTU (Needham et al., 2017). Therefore, when validating the putative MDMS against resequencing results, we treat similarity percentages higher than 94.5% as relevant because they may indicate sequences of the same genus (Yarza et al., 2014).

**Table 1:** Six representative sequences used for validation using re-sequencing by MiSeq Illumina with self-designed specific primers.

Seq ID	Original seq length	Amplified seq length	% similarity	F-primer	R-primer
R001	449	225	100	CGTAGGCGGTTTCTTAAGTTTTGA	ACTCGGGTTTCTAATCCTCTTCG
R003	449	271	98.15	AAAGCCTGATCCAGCCACAT	ACTCTCCTCTCCCTTCCTCT
A016	495	439	95.91	TCAGGGTGAACGCTGGTAAC	TCCACCGGTACAGTCAACCT
A054	469	392	100	GCAAGTCAAACCCCGCTTAT	CCGGTGCTATTTGCAGGAGT
A073	521	318	97.17	ACCGGATAGGATGGCTCTCT	CGTCAGGTACCGTCATACCAG
A080	498	458	100	GGCTCAGAATGAACGCTTGAAA	GCCAGGGCTTCTTCTTTAGGT

Only sequences with high levels of similarity (>95%) and 100% query cover are shown.

The MDMS were compared against the draft genomes that were generated from the metagenomic analyses of samples obtained from the natural aquifers and desert rocks. The metagenomics study of aquifers included nine samples with a sequencing depth of 120 million sequences, leading to the generation of a total of 106 consolidated bins (with a minimum completion of 50% and a maximum contamination of 10%). In parallel, the analysis of desert rocks involved eight samples with a sequencing depth of 181 million sequences, resulting in the identification of 45 bins. Nine of the draft genomes presented similarities to the MDMS higher than 96% (Table 2). The estimated level of completeness for those genomes ranged from 54.38 to 96.47%. 15 of the MDMS were present in the assembly results of the same samples (Supplementary Table S3). Finding only 9 matches corresponds to the discovery that ribosomal protein genes may be absent in over 20–40% of nearly complete metagenome-assembled genomes (Mise and Iwasaki, 2022).

**Table 2:** Bins (draft genomes) with high similarity (genus level) to the MDMS (blastn results) and bin information (completeness and contamination level).

Environment	MDMS seq ID	seqid	% similarity	Overlap length	Seq length	Node length	Completeness	Contamination	Size
Confined aquifers	A034	bin.2.permissive_NODE_252	100	370	514	2,362	83.51	0.959	1,528,181
	A010	bin.8.permissive_NODE_72	99.707	341	542	14,242	94.64	4.444	5,203,795
Unconfined aquifers	A020	bin.10.permissive_NODE_12	99.603	504	504	23,198	55.87	0.094	686,180
	A014	bin.40.orig_NODE_559	99.788	471	471	29,535	61.46	0	954,011
	A078	bin.32.strict_NODE_9	98.11	529	530	155,909	96.47	0.352	2,729,365
Biofilm from aquifers	A083	bin.7.orig_NODE_10041	96.603	471	471	2,996	54.38	0	529,863
	A054	bin.26.permissive_NODE_64	99.787	469	469	4,606	59.33	0.854	834,322
	A146	bin.34.permissive_NODE_1	99.656	291	558	52,977	56.38	0	621,998
Har Michya	R008	bin.15.strict_NODE_164	98.795	166	445	656	95.95	3.636	6,110,660

To ensure the integrity of the MDMS data, we performed an additional chimera check, specifically targeted to the 163 MDMS. Among the sequences analyzed, 20 sequences exhibited potential chimeric features (Supplementary Table S2). Although the low false-positive chimera detection was reported by DECIPHER (Firth et al., 2009), some of the 20 MDMS sequences which were suspected as chimeric were found to be similar to sequences in the metagenomic data in the validation process. Due to the limited overlap of the reads and low coverage percentages observed in some of the validated sequences, drawing definitive conclusions about the suspected chimeric sequences poses challenges. Thus, it is without doubt that several of the putative MDMS might be chimeric, which suggests that their taxonomic and phylogenetic analysis are incorrect.

**Figure 5:**
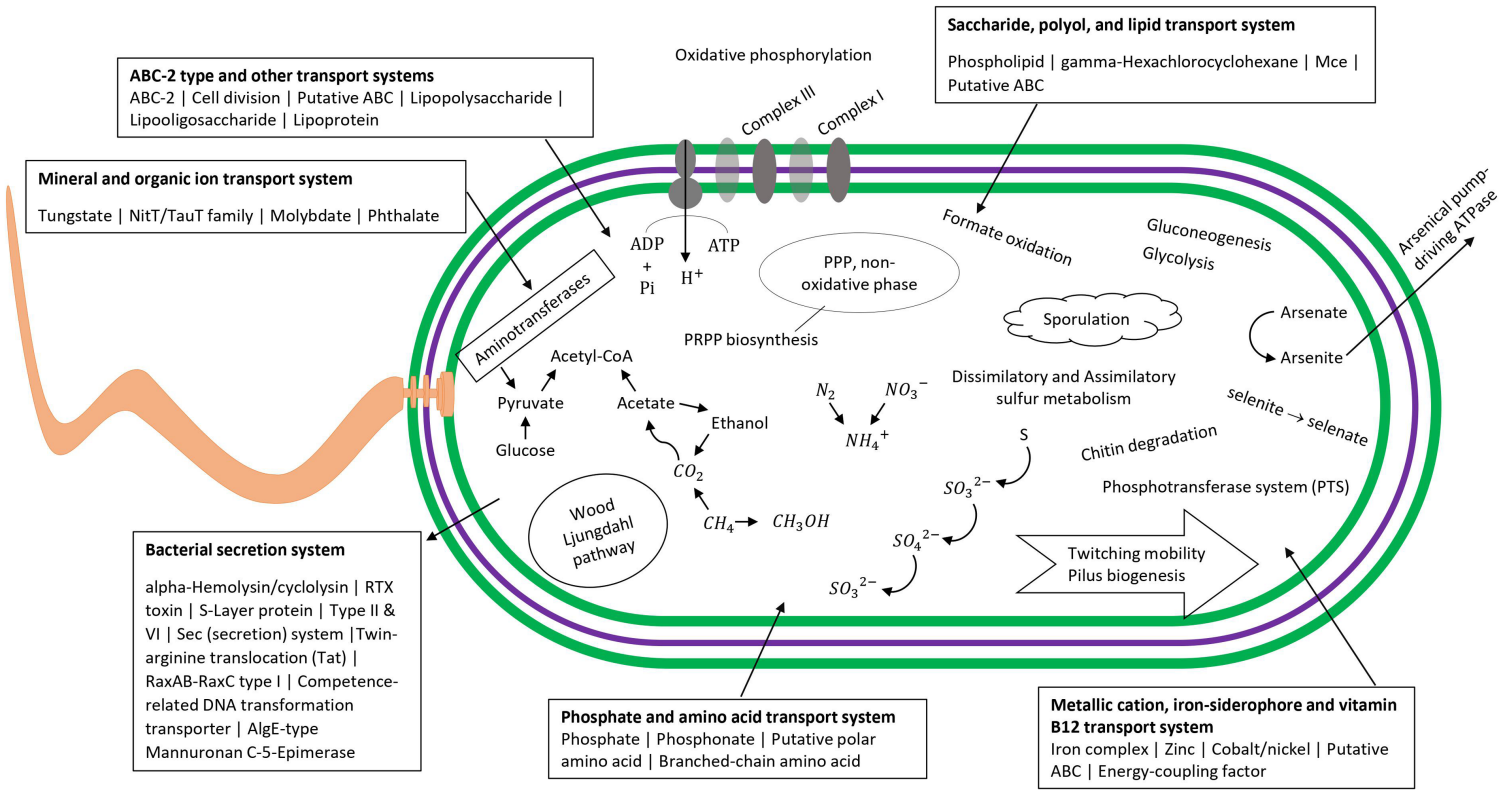
Visualized map of the metabolic capacities of the putative MDMS A010 based on METABOLIC results and Prokka. Information for other genomes is in Supplementary Table S4.

The nine draft genome matches to putative MDMS were characterized in terms of metabolic capacities based on their genes (Supplementary Table S4). Table 3 provides the assumed taxonomic attribution for the 9 MDMS. A034 is probably a new class within the Nitrospirota phylum, A010 is related to the Desulfuromonadota and it may be a new class within this phylum or a new, separate phylum. A078 and R008 belong to the Gammaproteobacteria and Chloroflexota, respectively. Five of the MDMS genomes were identified as part of the Patescibacteria super-phylum, such that A020 and A083 are apparently Paceibacteria, A014 is Microgenomatia, and A054 and A0146 are putative new candidate phyla. MDMS that were identified as part of the Patescibacteria super-phylum have fewer features than the other MDMS (Table 3 and Supplementary Table S4). Such a discrepancy could be caused by the typically small genome size, relatively small percentage of completeness, and lack of basic metabolic capacities that characterized the members of this group (Tian et al., 2020), but it could also be due to the lack of information about the functional genes of these uncultured microorganisms. Figure 5 presents some of the metabolic capacities of A010 (related to the Desulfuromonadota) and demonstrates the large amount of information that can be tapped about a prevalent MDMS (A010 constituted 40% of the reads in one sample) but that may be ignored due to their low similarity to existing databases. Bin A010 was assembled with a completion level of 94.6%. In addition to the comprehensive information about bacterial transport systems, we found genes whose expression controls morphology properties such as gram negativity, rod shape and basal body flagella. Moreover, it also contained genes for twitching mobility, sporulation, gluconeogenesis and glycolysis, chitin degradation, formate oxidation, selenate and arsenate reduction, and parts of the nitrogen and sulfur cycles. This metagenome-assembled genome also contained genes such as OmcS (outer-membrane hexaheme c-type cytochrome) and PilA (pilin monomers) that are typical in members of the Desulfuromonadota group and indicate their potential to transfer electrons extracellularly either to iron mineral particles or to microbial syntrophs, including methanogens (Elul et al., 2021). Given its origin from water aquifers, this bacterium could play a crucial part in carbon cycling and nutrient transformations within aquatic ecosystems.

**Table 3:** MDMS attribution to phyla based on 16S rRNA BLAST comparison to databases, location on the GTDB phylogenetic tree and information from the matchings with the draft genomes.

SeqID	Phylum
A034	Nitrospirota (new class)
A010	Desulfuromonadota (new class/phylum)
A020	Paceibacteria
A014	Microgenomatia
A078	Gammaproteobacteria
A083	Paceibacteria
A054	Putative new candidate phyla
A146	Putative new candidate phyla
R008	Chloroflexota

## 4. Conclusion

Microbial dark matter (MDM) comprises an immense diversity of yet-uncultivated bacteria. While cultivation independent techniques have been exploited in recent years to expand our knowledge about MDM, the bulk of microbial ecology studies continue to use 16S rRNA gene amplicon sequencing to characterize the microbial communities in a wide range of environments. When using this technique, researchers encounter groups of sequences that cannot be classified under known lineages in the existing databases, sequences that are now identified as belonging to the group of microbial dark matter sequences (MDMS). While these sequences are discarded from most analytical pipelines, they may still play important roles in environmental functioning. Furthermore, while in some well-studied environments, the ecological contribution of the MDMS may be negligible, their presence in the community in certain under-studied environments may be essential. Illuminating their functional contribution in these cases may facilitate a more robust and better understanding of the unique microbial community structures of these environments.

Here, in addition to demonstrating that microbial dark matter indeed present in amplicon sequencing, we present a pipeline to examine the MDM hidden in amplicon sequencing analysis. This study demonstrates that these abundant unidentified OTUs might be an essential part of their ecosystems. Therefore, we encourage researchers to retain these sequences and examine them as they might correspond to complete genomes containing metabolic functions critical to their ecosystems. Though they must be treated carefully, the results of MDMS investigations can be used to expand microbial databases and to situate these microorganisms in the tree of life, which together will promote a better comprehension of their evolution and contribute to the evolving taxonomy of the microbial world.

## Data availability statement

The datasets associated with this study have been deposited in the National Center for Biotechnology Information (NCBI) database. A comprehensive overview of these datasets, including their corresponding accession numbers and types, is provided in Supplementary Table S5.

## Author contributions

HB and NF implemented all bioinformatic analyses and wrote the main manuscript text. HB prepared all figures. IN and ML-N performed samples of rocks and aquifers samples collection and DNA extraction. AK and AS supervised the project. All authors reviewed the manuscript.

## Footnotes

^1^http://qiime.org/

## In memoriam

This paper is dedicated to the memory of Prof. Alex Sivan, who participated in this study.
