# Supplementary material for: Microbial dark matter sequences verification in amplicon sequencing and environmental metagenomics data
Source: Front Microbiol. 2023 Nov 2;14:1247119. doi: 10.3389/fmicb.2023.1247119 (PMC10656735; doi:10.3389/fmicb.2023.1247119)
Supplement: Supplementary file 1 [file Data_Sheet_1.docx]

Supplementary Material

**Microbial Dark Matter Sequences Verification in Amplicon Sequencing and Environmental Metagenomics Data**

**Hana Barak, Naomi Fuchs, Michal Liddor-Naim, Irit Nir, Alex Sivan and Ariel Kushmaro^*^**

***** **Correspondence**: Ariel Kushmaro, [arielkus@bgu.ac.il](mailto:arielkus@bgu.ac.il)


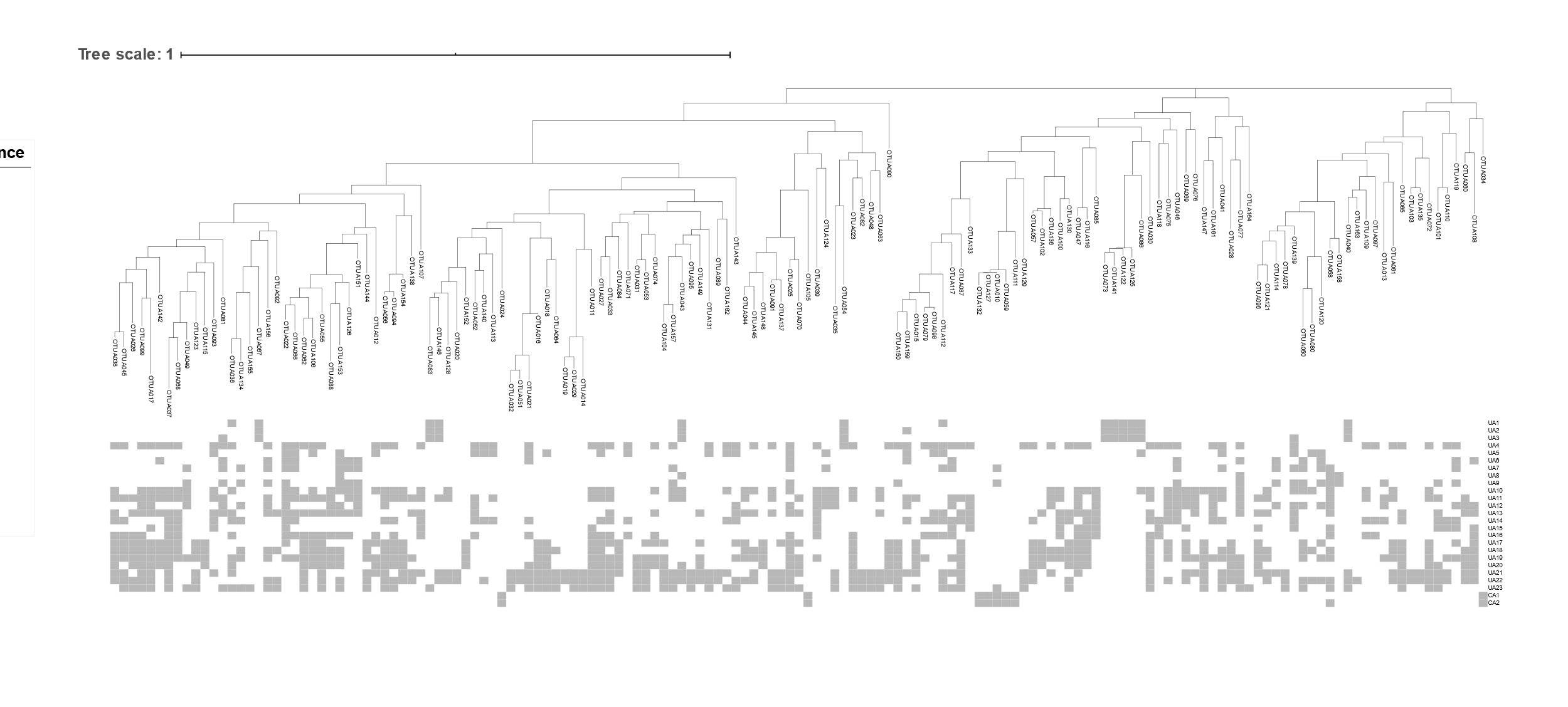


A


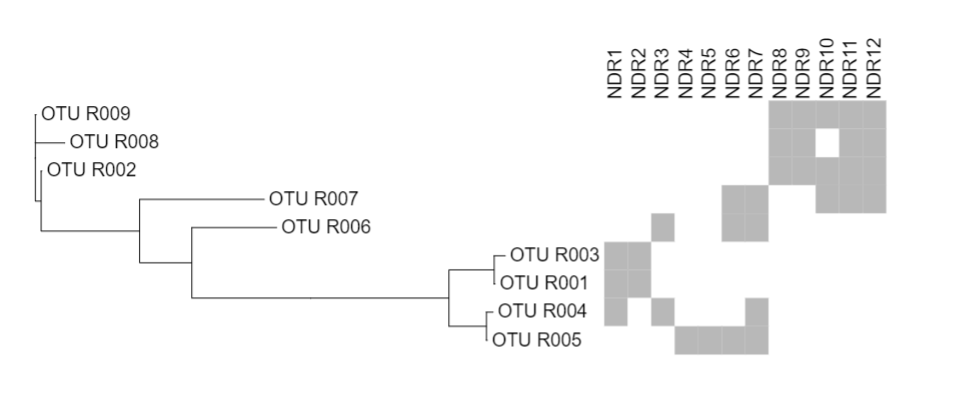


B

**Supplementary Figure 1**: Heatmaps of (A) Arava valley aquifers and (B) Negev desert rocks. The OTUs are ordered by phylogenetic group. Gray and white squares represent the presence and absence, respectively, of the OTUs in each sample. NDR – Negev desert rock samples; UA – unconfined aquifer samples; CA – confined aquifer samples. OTUs are marked with R for Rocks or A for Aquifers. OTU branches related to Nitrospirota phylum according to phylogenetic tree in Figure 3 are highlighted.


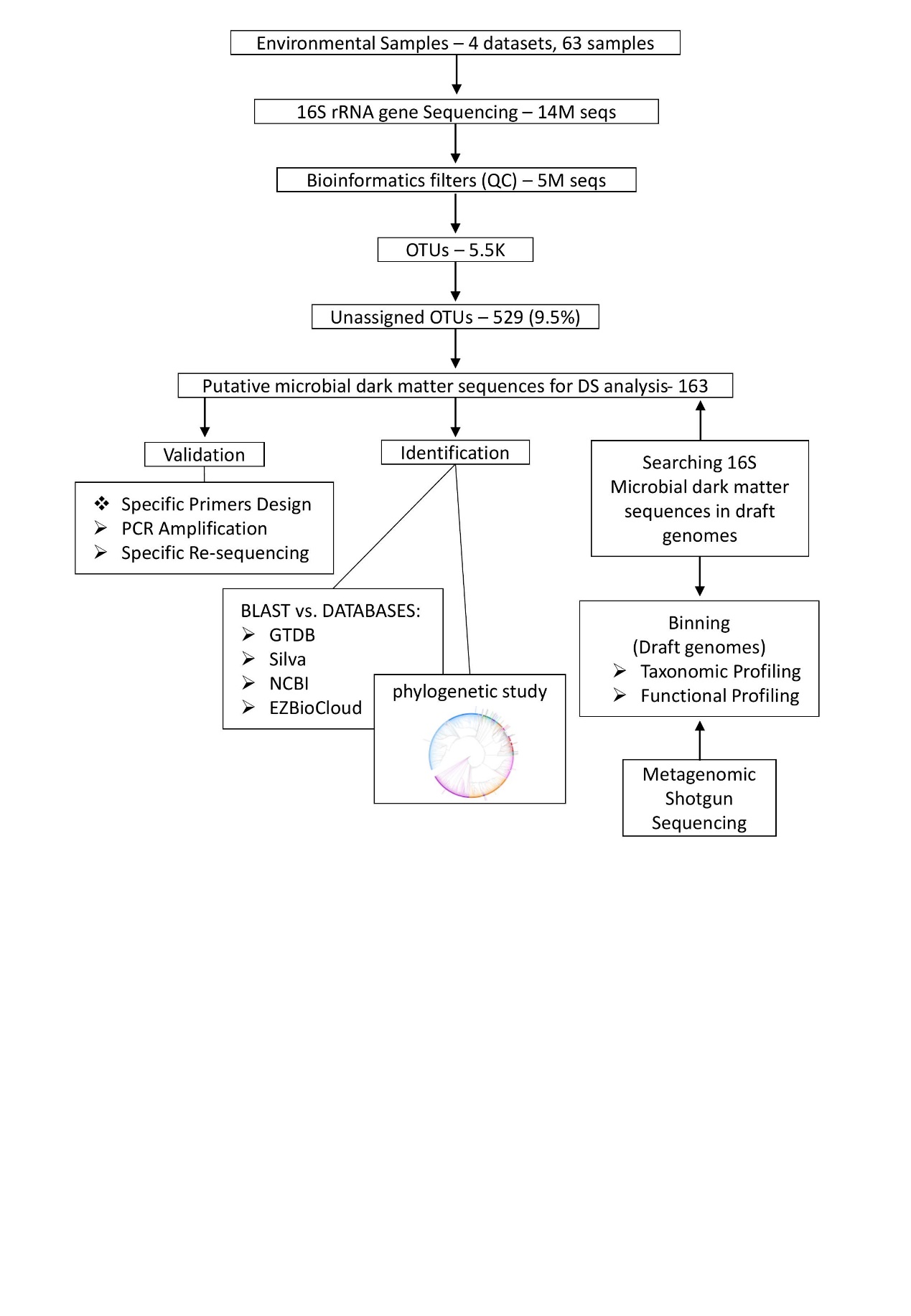


**Supplementary Figure 2**: A concise representation of the methodology pipeline adopted in this study.

**Supplementary Table 2**: This table provides an overview of sequences within our dataset that have been flagged as potentially chimeric using the DECIPHER tool.

| OTU_A164 | OTU_A123 | OTU_A096 | OTU_A062 |
| --- | --- | --- | --- |
| OTU_A163 | OTU_A114 | OTU_A091 | OTU_A058 |
| OTU_A148 | OTU_R005 | OTU_R004 | OTU_A039 |
| OTU_A138 | OTU_A105 | OTU_A083 | OTU_A025 |
| OTU_A130 | OTU_A100 | OTU_A082 | OTU_A012 |

**Supplementary Table 3**: Scaffolds (assembly results) that show high similarity (genus level) to the MDMS.

| Environment | Seq ID | Assembly Node ID | % Similarity | Overlap Length | Seq Length | Node Length |
| --- | --- | --- | --- | --- | --- | --- |
| Negev Desert Rocks | R001 | NODE_29774 | 99.312 | 436 | 449 | 1457 |
|  | R003 | NODE_29774 | 97.706 | 436 | 449 | 1457 |
|  | R005 | NODE_47404 | 98.844 | 519 | 586 | 1039 |
| Confined Aquifers | A010 | NODE_2431 | 100 | 542 | 542 | 5428 |
|  | A024 | NODE_10581 | 99.174 | 484 | 484 | 1013 |
|  | A034 | NODE_2526 | 100 | 363 | 514 | 5215 |
| Unconfined Aquifers | A030 | NODE_63024 | 100 | 541 | 541 | 1351 |
|  | A014 | NODE_559 | 99.788 | 471 | 471 | 29535 |
|  | A011 | NODE_100851 | 99.641 | 557 | 557 | 1036 |
|  | A035 | NODE_24540 | 99.616 | 521 | 521 | 2335 |
|  | A020 | NODE_824 | 99.603 | 504 | 504 | 23123 |
|  | A018 | NODE_40799 | 98.488 | 463 | 481 | 1732 |
|  | A078 | NODE_51 | 98.148 | 432 | 530 | 126879 |
|  | A083 | NODE_10041 | 96.603 | 471 | 470 | 2996 |
|  | A054 | NODE_7290 | 99.36 | 469 | 469 | 3930 |

**Supplementary Table 4**: MDMS attribution to phyla and representative features based on the matching draft genomes.

| SeqID | Features |
| --- | --- |
| A034 | **HMMHit** • Aminotransferase • Fatty acid degradation • Phenol → Benzoyl-CoA • Chitin degrading • Pyruvate oxidation • Formate oxidation • Acetate to acetyl-CoA • Reductive acetyl-CoA pathway (Wood-Ljungdahl pathway) • Sulfite reduction • Sulfur oxidation • Sulfate reduction • Dissimilatory and Assimilatory sulfur metabolism • DMSO metabolism • Metabolism of organic sulfur • Sulfur-related amino acid metabolism • Arsenate reduction • Ni-Fe Hydrogenase • Oxidative phosphorylation (Complex I, Complex V) •  **KEGGModuleHit** • ABC-2 type and other transport systems: ABC-2\| Lipoprotein-releasing \| Cell division \| Putative ABC \| Lipopolysaccharide export • Aminoacyl-tRNA biosynthesis • Ornithine biosynthesis (glutamate → ornithine) • Shikimate pathway, phosphoenolpyruvate + erythrose-4P → chorismite • Tryptophan biosynthesis, chorismate → tryptophan • ATP synthesis (NADH:quinone oxidoreductase\|F-type ATPase) • Bacterial secretion system: alpha-Hemolysin/cyclolysin \| RTX toxin \| S-Layer protein \|Type II general secretion pathway \|Sec (secretion)\| Twin-arginine translocation (Tat) \| RaxAB-RaxC type\| Competence-related DNA transformation\| AlgE-type Mannuronan C-5-Epimerase • Valine/isoleucine biosynthesis, pyruvate → valine / 2-oxobutanoate→isoleucine • Isoleucine biosynthesis, threonine → 2-oxobutanoate → isoleucine • Central carbohydrate metabolism: Glycolysis, core module involving three-carbon compounds \| Gluconeogenesis, oxaloacetate → fructose-6P \| PRPP biosynthesis, ribose 5P → PRPP \| Pentose phosphate pathway, non-oxidative phase, fructose 6P → ribose 5P \| Pyruvate oxidation, pyruvate → acetyl-CoA • Cofactor and vitamin metabolism: NAD biosynthesis, aspartate → NAD \| Pantothenate biosynthesis, valine/L-aspartate → pantothenate \| Heme biosynthesis, glutamate → protoheme/siroheme \| Cobalamin biosynthesis, cobinamide → cobalamin \| Biotin biosynthesis, pimeloyl-ACP/CoA → biotin \| Biotin biosynthesis, BioI pathway, long-chain-acyl-ACP → pimeloyl-ACP → biotin \| Biotin biosynthesis, BioW pathway, pimelate → pimeloyl-CoA → biotin • Cysteine biosynthesis, serine → cysteine • DNA polymerase III complex • Drug efflux transporter/pump: Multidrug resistance, efflux pump VexEF-TolC • Fatty acid biosynthesis and degradation: beta-Oxidation, acyl-CoA synthesis • Histidine biosynthesis, PRPP → histidine • Lipid metabolism: Phosphatidylethanolamine (PE) biosynthesis, PA → PS → PE • Lipopolysaccharide metabolism: Lipopolysaccharide biosynthesis, KDO2-lipid A \| CMP-KDO biosynthesis • Metallic cation, iron-siderophore and vitamin B12 transport system: Iron complex transport system \| Zinc transport system \| Cobalt/nickel transport system\|Nickel transport system • Mineral and organic ion transport system: Tungstate transport system \| Phthalate transport system • Nucleotide sugar biosynthesis • Peptide and nickel transport system: Peptides/nickel transport system \| Oligopeptide transport system • Phosphate and amino acid transport system: Phosphate transport system\| Phosphonate transport system \| Branched-chain amino acid transport system • Polyamine biosynthesis, arginine → agmatine → putrescine → spermidine • Protein processing: p97-Ufd1-Npl4 complex\| HRD1/SEL1 ERAD complex • Purine metabolism: Adenine ribonucleotide biosynthesis, IMP → ADP,ATP \| Guanine ribonucleotide biosynthesis IMP → GDP,GTP • Pyrimidine metabolism: Uridine monophosphate biosynthesis, glutamine (+ PRPP) → UMP \| Pyrimidine ribonucleotide biosynthesis, UMP → UDP/UTP,CDP/CTP • Ribosome • RNA polymerase • RNA degradosome • Saccharide, polyol, and lipid transport system: Phospholipid transport system \| gamma-Hexachlorocyclohexane transport system \| Mce transport system • Threonine biosynthesis, aspartate → homoserine → threonine • Sulfur metabolism: Dissimilatory sulfate reduction, sulfate → H2S • |
| A010 | **HMMHit** • Aminotransferase • Fatty acid degradation • Phenol → Benzoyl-CoA • Amylolytic enzymes • Chitin degrading • Pyruvate oxidation • Acetogenesis • Formate oxidation • Methane oxidation • Reductive acetyl-CoA pathway (Wood-Ljungdahl pathway) • N2 fixation • Nitrite reduction to ammonia • Sulfur oxidation • Sulfate reduction • Dissimilatory and Assimilatory sulfur metabolism • DMSO metabolism • Metabolism of organic sulfur • Sulfur-related amino acid metabolism • Arsenate reduction • Selenate reduction • Ni-Fe Hydrogenase • Oxidative phosphorylation (Complex I, Complex V) •  **KEGGModuleHit** • ABC-2 type and other transport systems: Lipopolysaccharide\| Lipooligosaccharide\|ABC-2\|Lipoprotein-releasing\|Cell division\|Putative ABC\|Lipopolysaccharide export • Aminoacyl-tRNA biosynthesis • Proline biosynthesis, glutamate → proline • Ornithine biosynthesis (glutamate → ornithine) • Tryptophan biosynthesis, chorismate → tryptophan • ATP synthesis: NADH:quinone oxidoreductase\|F-type ATPase\| V/A-type ATPase\| Cytochrome b6f complex • Bacterial secretion system: alpha-Hemolysin/cyclolysin \| RTX toxin \| S-Layer protein \|Type II general secretion pathway\| Type VI \|Sec (secretion)\| Twin-arginine translocation (Tat) \| RaxAB-RaxC type\| Competence-related DNA transformation\| AlgE-type Mannuronan C-5-Epimerase • Valine/isoleucine biosynthesis, pyruvate → valine / 2-oxobutanoate→isoleucine • Leucine biosynthesis, 2-oxoisovalerate → 2-oxoisocaproate • Isoleucine biosynthesis, threonine → 2-oxobutanoate → isoleucine • Carbon fixation: Reductive acetyl-CoA pathway, Wood-Ljungdahl pathway • Central carbohydrate metabolism: Glycolysis (Embden-Meyerhof pathway), glucose → pyruvate\| Glycolysis, core module involving three-carbon compounds \| Gluconeogenesis, oxaloacetate → fructose-6P \| PRPP biosynthesis, ribose 5P → PRPP \| Pentose phosphate pathway, non-oxidative phase, fructose 6P → ribose 5P \| Pyruvate oxidation, pyruvate → acetyl-CoA •Cofactor and vitamin metabolism:  NAD biosynthesis, aspartate → NAD \| Pantothenate biosynthesis, valine/L-aspartate → pantothenate \| Coenzyme A biosynthesis, pantothenate → CoA \| Cobalamin biosynthesis, cobinamide → cobalamin \| Biotin biosynthesis, pimeloyl-ACP/CoA → biotin \| Thiamine biosynthesis, AIR → thiamine-P/thiamine-2P \| C1-unit interconversion, prokaryotes \| Pimeloyl-ACP biosynthesis, BioC-BioH pathway, malonyl-ACP → pimeloyl-ACP \| Biotin biosynthesis, BioI pathway, long-chain-acyl-ACP → pimeloyl-ACP → biotin \| Biotin biosynthesis, BioW pathway, pimelate → pimeloyl-CoA → biotin • Cysteine biosynthesis, serine → cysteine • Methionine degradation • DNA polymerase III complex • Drug efflux transporter/pump: Multidrug resistance, MdlAB/SmdAB transporter \| Multidrug resistance, efflux pump VexEF-TolC • Fatty acid biosynthesis and degradation: Fatty acid biosynthesis, initiation \| Fatty acid biosynthesis, elongation • Lipid metabolism: Phosphatidylethanolamine (PE) biosynthesis, PA → PS → PE • Lipopolysaccharide metabolism: Lipopolysaccharide biosynthesis, KDO2-lipid A \| CMP-KDO biosynthesis \| ADP-L-glycero-D-manno-heptose biosynthesis • Lysine metabolism: Lysine biosynthesis, succinyl-DAP pathway, aspartate → lysine \| Lysine biosynthesis, DAP dehydrogenase pathway, aspartate → lysine \| Lysine biosynthesis, DAP aminotransferase pathway, aspartate → lysine • Metallic cation, iron-siderophore and vitamin B12 transport system: Iron complex transport system \| Zinc transport system \| Cobalt/nickel transport system\|Nickel transport system \| Putative ABC transport system \| Energy-coupling factor transport system • Mineral and organic ion transport system: Tungstate transport system \| NitT/TauT family transport system \| Molybdate transport system \| Phthalate transport system \| Nitrate/nitrite transport system • Nitrogen metabolism: Nitrogen fixation, nitrogen → ammonia • Nucleotide sugar biosynthesis • Other carbohydrate metabolism: Trehalose biosynthesis, D-glucose 1P → trehalose • Peptide and nickel transport system: Peptides/nickel transport system • Phosphate and amino acid transport system: Phosphate transport system\| Phosphonate transport system \| Putative polar amino acid transport system \| Branched-chain amino acid transport system • Phosphotransferase system (PTS): PTS system, fructose-specific II component \| PTS system, mannose-specific II component • Polyamine biosynthesis, arginine → agmatine → putrescine → spermidine • Polyketide sugar unit biosynthesis: dTDP-L-rhamnose biosynthesis • Purine metabolism: Adenine ribonucleotide biosynthesis, IMP → ADP,ATP \| Guanine ribonucleotide biosynthesis IMP → GDP,GTP • Pyrimidine metabolism: Uridine monophosphate biosynthesis, glutamine (+ PRPP) → UMP \| Pyrimidine ribonucleotide biosynthesis, UMP → UDP/UTP,CDP/CTP \| Pyrimidine deoxyribonuleotide biosynthesis, CDP/CTP → dCDP/dCTP,dTDP/dTTP • Ribosome • RNA polymerase • RNA degradosome • Saccharide, polyol, and lipid transport system: Phospholipid transport system \| Putative ABC transport system \| gamma-Hexachlorocyclohexane transport system \| Mce transport system • Threonine biosynthesis, aspartate → homoserine → threonine • Terpenoid backbone biosynthesis: C5 isoprenoid biosynthesis, non-mevalonate pathway \| C10-C20 isoprenoid biosynthesis • Two-component regulatory system:  NtrY-NtrX (nitrogen regulation) two-component regulatory system \| HydH-HydG (metal tolerance) two-component regulatory system \| PilS-PilR (type 4 fimbriae synthesis) two-component regulatory system \| CheA-CheYBV (chemotaxis) two-component regulatory system • |
| A020 | **HMMHit** • Amylolytic enzymes •  **KEGGModuleHit** • ABC-2 type and other transport systems: ABC-2\|Cell division \|Putative ABC • Aminoacyl-tRNA biosynthesis • Bacterial secretion system: S-Layer protein \|Type II general secretion pathway\| Sec (secretion)\|Competence-related DNA transformation • DNA polymerase III complex • Mineral and organic ion transport system: Phthalate transport system • Peptide and nickel transport system: Peptides/nickel transport system • Phosphate and amino acid transport system: Branched-chain amino acid transport system • Polyamine biosynthesis, arginine → agmatine → putrescine → spermidine • Pyrimidine metabolism: Pyrimidine deoxyribonuleotide biosynthesis, CDP/CTP → dCDP/dCTP,dTDP/dTTP • Ribosome • RNA polymerase • |
| A014 | **HMMHit** • Metabolism of organic sulfur • Oxidative phosphorylation (Complex II, Complex V) •  **KEGGModuleHit** • ABC-2 type and other transport systems: ABC-2\| Cell division \| Putative ABC • Aminoacyl-tRNA biosynthesis • ATP synthesis: F-type ATPase • Bacterial secretion system: S-Layer protein \|Type II general secretion pathway\| Sec (secretion)\|Competence-related DNA transformation • Central carbohydrate metabolism: PRPP biosynthesis, ribose 5P → PRPP • DNA polymerase III complex • Mineral and organic ion transport system: Phthalate transport system • Polyketide sugar unit biosynthesis: dTDP-L-rhamnose biosynthesis • Pyrimidine metabolism: Pyrimidine deoxyribonuleotide biosynthesis, CDP/CTP → dCDP/dCTP,dTDP/dTTP • Ribosome • RNA polymerase • RNA degradosome • Terpenoid backbone biosynthesis: C5 isoprenoid biosynthesis, mevalonate pathway • |
| A078 | **HMMHit** • Aminotransferase • Fatty acid degradation • Phenol → Benzoyl-CoA • Chitin degrading • Acetate to acetyl-CoA • CBB cycle – Rubisco • Nitrate reduction • Nitrite reduction to ammonia • Sulfur oxidation • Sulfate reduction • Dissimilatory and Assimilatory sulfur metabolism • DMSO metabolism • Metabolism of organic sulfur • Sulfur-related amino acid metabolism • Urease • Arsenate reduction • Ni-Fe Hydrogenase • Oxidative phosphorylation (Complex I, Complex II, Complex III, Complex IV, Complex V) •  **KEGGModuleHit** • ABC-2 type and other transport systems: Lipopolysaccharide \| ABC-2 \| Lipoprotein-releasing \| Cell division \| Putative ABC \| Heme \|Lipopolysaccharide export • Aminoacyl-tRNA biosynthesis • Proline biosynthesis, glutamate → proline • Ornithine biosynthesis (glutamate → ornithine) • Shikimate pathway, phosphoenolpyruvate + erythrose-4P → chorismite • Tryptophan biosynthesis, chorismate → tryptophan • Cymene degradation, p-cymene → p-cumate • ATP synthesis: NADH:quinone oxidoreductase\| Succinate dehydrogenase\| Cytochrome bc1 complex respiratory unit\| Cytochrome bc1 complex\| Cytochrome c oxidase, cbb3-type\|F-type ATPase • Bacterial secretion system: alpha-Hemolysin/cyclolysin \| RTX toxin \| S-Layer protein \| Adhesin protein\|Type II general secretion pathway \|Sec (secretion)\| Twin-arginine translocation (Tat) \| RaxAB-RaxC type\| Competence-related DNA transformation\| AlgE-type Mannuronan C-5-Epimerase • Valine/isoleucine biosynthesis, pyruvate → valine / 2-oxobutanoate→isoleucine • Leucine biosynthesis, 2-oxoisovalerate → 2-oxoisocaproate • Isoleucine biosynthesis, threonine → 2-oxobutanoate → isoleucine • Carbon fixation (Reductive pentose phosphate cycles: Calvin cycle, ribulose-5P → glyceraldehyde-3P, glyceraldehyde-3P → ribulose-5P \| CAM (Crassulacean acid metabolism), dark) • Central carbohydrate metabolism: Glycolysis (Embden-Meyerhof pathway), glucose → pyruvate \| Glycolysis, core module involving three-carbon compounds \| Gluconeogenesis, oxaloacetate → fructose-6P \| Pentose phosphate pathway (Pentose phosphate cycle) \| PRPP biosynthesis, ribose 5P → PRPP \| Pentose phosphate pathway, oxidative phase, glucose 6P → ribulose 5P \| Pentose phosphate pathway, non-oxidative phase, fructose 6P → ribose 5P \| Citrate cycle (TCA cycle, Krebs cycle) \| Citrate cycle, first carbon oxidation, oxaloacetate → 2-oxoglutarate \| Citrate cycle, second carbon oxidation, 2-oxoglutarate → oxaloacetate \| Pyruvate oxidation, pyruvate → acetyl-CoA • Cofactor and vitamin metabolism: NAD biosynthesis, aspartate → NAD \| Ubiquinone biosynthesis, prokaryotes, chorismate → ubiquinone \| Pantothenate biosynthesis, valine/L-aspartate → pantothenate \| Coenzyme A biosynthesis, pantothenate → CoA \| Heme biosynthesis, glutamate → protoheme/siroheme \| Biotin biosynthesis, pimeloyl-ACP/CoA → biotin \| Pimeloyl-ACP biosynthesis, BioC-BioH pathway, malonyl-ACP → pimeloyl-ACP \| Biotin biosynthesis, BioI pathway, long-chain-acyl-ACP → pimeloyl-ACP→ biotin \| Biotin biosynthesis, BioW pathway, pimelate → pimeloyl-CoA → biotin • Cysteine biosynthesis, serine → cysteine • DNA polymerase III complex • Drug efflux transporter/pump: Macrolide resistance, MacAB-TolC transporter \| Multidrug resistance, efflux pump VexEF-TolC • Fatty acid biosynthesis and degradation: Fatty acid biosynthesis, initiation \| Fatty acid biosynthesis, elongation \| beta-Oxidation, acyl-CoA synthesis • Histidine biosynthesis, PRPP → histidine • Lipid metabolism: Phosphatidylethanolamine (PE) biosynthesis, PA → PS → PE • Lipopolysaccharide metabolism: Lipopolysaccharide biosynthesis, KDO2-lipid A \| CMP-KDO biosynthesis \| ADP-L-glycero-D-manno-heptose biosynthesis • Lysine metabolism: Lysine biosynthesis, succinyl-DAP pathway, aspartate → lysine \| Lysine biosynthesis, DAP dehydrogenase pathway, aspartate → lysine \| Lysine biosynthesis, DAP aminotransferase pathway, aspartate → lysine • Metallic cation, iron-siderophore and vitamin B12 transport system: Iron complex transport system \| Cobalt/nickel transport system\|Nickel transport system \| Putative ABC transport system • Mineral and organic ion transport system: NitT/TauT family transport system \| Iron(III) transport system \| Phthalate transport system \| Nitrate/nitrite transport system • Nitrogen metabolism: Dissimilatory nitrate reduction, nitrate → ammonia • Peptide and nickel transport system: Peptides/nickel transport system • Phosphate and amino acid transport system: Phosphate transport system\| Branched-chain amino acid transport system \| Urea transport system • Polyketide sugar unit biosynthesis: dTDP-L-rhamnose biosynthesis • Purine metabolism: Inosine monophosphate biosynthesis, PRPP + glutamine → IMP \| Adenine ribonucleotide biosynthesis, IMP → ADP,ATP \| Guanine ribonucleotide biosynthesis IMP → GDP,GTP • Pyrimidine metabolism: Uridine monophosphate biosynthesis, glutamine (+ PRPP) → UMP \| Pyrimidine ribonucleotide biosynthesis, UMP → UDP/UTP,CDP/CTP \| Pyrimidine deoxyribonuleotide biosynthesis, CDP/CTP → dCDP/dCTP,dTDP/dTTP • Ribosome • RNA polymerase • RNA degradosome • Saccharide, polyol, and lipid transport system: Phospholipid transport system \| Putative ABC transport system \| gamma-Hexachlorocyclohexane transport system \| Mce transport system • Threonine biosynthesis, aspartate → homoserine → threonine • Serine biosynthesis, glycerate-3P → serine • Terpenoid backbone biosynthesis: C5 isoprenoid biosynthesis, non-mevalonate pathway • Two-component regulatory system:  PhoR-PhoB (phosphate starvation response) two-component regulatory system \| PhoQ-PhoP (magnesium transport) two-component regulatory system \| QseC-QseB (quorum sensing) two-component regulatory system \| AlgZ-AlgR (alginate production) two-component regulatory system \| GlnL-GlnG (nitrogen regulation) two-component regulatory system \| NtrY-NtrX (nitrogen regulation) two-component regulatory system \| PilS-PilR (type 4 fimbriae synthesis) two-component regulatory system \| WspE-WspRF (chemosensory) two-component regulatory system \| FlrB-FlrC (polar flagellar synthesis) two-component regulatory system \| RegB-RegA (redox response) two-component regulatory system • |
| A083 | **HMMHit** • Nitrite reduction • Oxidative phosphorylation (Complex V) •  **KEGGModuleHit** • ABC-2 type and other transport systems: ABC-2\| Cell division • Aminoacyl-tRNA biosynthesis • ATP synthesis: F-type ATPase • Bacterial secretion system: S-Layer protein \|Type II general secretion pathway\| Sec (secretion) \| Competence-related DNA transformation • DNA polymerase III complex • Mineral and organic ion transport system: Phthalate transport system • Peptide and nickel transport system: Peptides/nickel transport system • Ribosome • RNA polymerase • RNA degradosome • |
| A054 | **HMMHit** • Endohemicellulases • Acetogenesis • Oxidative phosphorylation (Complex V) •  **KEGGModuleHit** • ABC-2 type and other transport systems: ABC-2 \| Cell division \| Putative ABC • ATP synthesis: F-type ATPase • Bacterial secretion system: S-Layer protein \|Type II general secretion pathway\| Sec (secretion)\|Competence-related DNA transformation • DNA polymerase III complex • Mineral and organic ion transport system: Phthalate transport system • Pyrimidine metabolism: Pyrimidine deoxyribonuleotide biosynthesis, CDP/CTP → dCDP/dCTP,dTDP/dTTP • Ribosome • RNA polymerase • RNA degradosome • |
| A146 | •  **KEGGModuleHit** • ABC-2 type and other transport systems: Lipopolysaccharide \| ABC-2 \| Cell division \| Putative ABC • Bacterial secretion system: S-Layer protein \|Type II general secretion pathway\| Sec (secretion)\|Competence-related DNA transformation • DNA polymerase III complex • Mineral and organic ion transport system: Phthalate transport system • Phosphate and amino acid transport system: Branched-chain amino acid transport system • Ribosome • RNA polymerase • RNA degradosome • |
| R008 | **HMMHit** • Aminotransferase • Fatty acid degradation • Cellulose degrading • Hemicullulose debranching • Amylolytic enzymes • Chitin degrading • Lactate utilization • Acetogenesis • Acetate to acetyl-CoA • Aerobic CO oxidation • Formate oxidation • Sulfate reduction • Dissimilatory and Assimilatory sulfur metabolism • DMSO metabolism • Metabolism of organic sulfur • Sulfur-related amino acid metabolism • Chlorite reduction • Arsenate reduction • Oxidative phosphorylation (Complex I, Complex II, Complex III, Complex IV, Complex V) •  **KEGGModuleHit** • ABC-2 type and other transport systems: Fluoroquinolone\| Lipopolysaccharide\| ABC-2 \| Putative ABC \| Heme • Aminoacyl-tRNA biosynthesis • Proline biosynthesis, glutamate → proline • Ornithine biosynthesis (glutamate → ornithine) • Urea cycle • Ornithine biosynthesis, mediated by LysW, glutamate → ornithine • Shikimate pathway, phosphoenolpyruvate + erythrose-4P → chorismite • Tryptophan biosynthesis, chorismate → tryptophan • ATP synthesis: NADH:quinone oxidoreductase\| Succinate dehydrogenase\| Fumarate reductase\| Cytochrome bc1 complex respiratory unit\| Cytochrome bc1 complex\| Cytochrome c oxidase\|F-type ATPase\| Cytochrome b6f complex • Bacterial secretion system: S-Layer protein \| Type IV\| Type VI \|Sec (secretion)\| Twin-arginine translocation (Tat) \| RaxAB-RaxC type\| Competence-related DNA transformation • Leucine degradation, leucine → acetoacetate + acetyl-CoA • Carbon fixation: Reductive pentose phosphate cycles: glyceraldehyde-3P → ribulose-5P \| CAM (Crassulacean acid metabolism), dark) \| Reductive citrate cycle (Arnon-Buchanan cycle) • Central carbohydrate metabolism: Glycolysis (Embden-Meyerhof pathway), glucose → pyruvate \| Glycolysis, core module involving three-carbon compounds \| Gluconeogenesis, oxaloacetate → fructose-6P \| Pentose phosphate pathway (Pentose phosphate cycle) \| PRPP biosynthesis, ribose 5P → PRPP \| Pentose phosphate pathway, non-oxidative phase, fructose 6P → ribose 5P \| Citrate cycle (TCA cycle, Krebs cycle) \| Citrate cycle, first carbon oxidation, oxaloacetate → 2-oxoglutarate \| Citrate cycle, second carbon oxidation, 2-oxoglutarate → oxaloacetate \| Pyruvate oxidation, pyruvate → acetyl-CoA \| Semi-phosphorylative Entner-Doudoroff pathway, gluconate → glycerate-3P• Cofactor and vitamin metabolism: Coenzyme A biosynthesis, pantothenate → CoA • Cysteine biosynthesis, serine → cysteine • Methionine degradation • Cysteine biosynthesis, homocysteine + serine → cysteine • DNA polymerase III complex • Drug efflux transporter/pump: Multidrug resistance, MdlAB/SmdAB transporter • Fatty acid biosynthesis and degradation: Fatty acid biosynthesis, initiation \| Fatty acid biosynthesis, elongation \| beta-Oxidation, acyl-CoA synthesis \| beta-Oxidation • Histidine biosynthesis, PRPP → histidine • Histidine degradation, histidine → N-formiminoglutamate → glutamate • Lipid metabolism: Ketone body biosynthesis, acetyl-CoA → acetoacetate/3-hydroxybutyrate/acetone \| Phosphatidylethanolamine (PE) biosynthesis, PA → PS → PE • Lysine metabolism: Lysine biosynthesis, mediated by LysW, 2-aminoadipate → lysine • Macrolide biosynthesis: Avermectin biosynthesis, 2-methylbutanoyl-CoA/isobutyryl-CoA → 6,8a-Seco-6,8a-deoxy-5-oxoavermectin 1a/1b aglycone → avermectin A1a/B1a/A1b/B1b • Metallic cation, iron-siderophore and vitamin B12 transport system: Iron complex transport system \| Putative zinc/manganese transport system\| Cobalt/nickel transport system \| Nickel transport system \| Energy-coupling factor transport system • Mineral and organic ion transport system: Sulfate transport system \| Tungstate transport system \| NitT/TauT family transport system \| Molybdate transport system \| Iron(III) transport system \| Thiamine transport system \| Osmoprotectant transport system \| Molybdate/tungstate transport system \| Phthalate transport system • Nucleotide sugar biosynthesis • Other carbohydrate metabolism: Glyoxylate cycle \| Nucleotide sugar biosynthesis, galactose → UDP-galactose \| Trehalose biosynthesis, D-glucose 1P → trehalose \| Galactose degradation, Leloir pathway, galactose → alpha-D-glucose-1P \| Propanoyl-CoA metabolism, propanoyl-CoA → succinyl-CoA • Peptide and nickel transport system: Peptides/nickel transport system \| Oligopeptide transport system • Phosphate and amino acid transport system: Phosphate transport system\| Putative glutamine transport system\| Putative polar amino acid transport system \| Branched-chain amino acid transport system • Phosphotransferase system (PTS): PTS system, fructose-specific II component • Polyketide sugar unit biosynthesis: dTDP-L-rhamnose biosynthesis • Purine metabolism: Inosine monophosphate biosynthesis, PRPP + glutamine → IMP \| Adenine ribonucleotide biosynthesis, IMP → ADP,ATP \| Guanine ribonucleotide biosynthesis IMP → GDP,GTP \| Purine degradation, xanthine → urea • Pyrimidine metabolism: Pyrimidine ribonucleotide biosynthesis, UMP → UDP/UTP,CDP/CTP • Repair system: Holo-TFIIH complex • Ribosome • RNA polymerase • RNA degradosome • Saccharide, polyol, and lipid transport system: Maltose/maltodextrin transport system \| Raffinose/stachyose/melibiose transport system \| Putative fructooligosaccharide transport system \| Putative sorbitol/mannitol transport system \| alpha-Glucoside transport system \| Cellobiose transport system \| Putative multiple sugar transport system \| Ribose transport system \| Putative simple sugar transport system \| arabinogalactan oligomer/maltooligosaccharide transport system \| Arabinosaccharide transport system \| Glucose/mannose transport system \| N,N'-Diacetylchitobiose transport system • Threonine biosynthesis, aspartate → homoserine → threonine • Two-component regulatory system: DesK-DesR (membrane lipid fluidity regulation) two-component regulatory system • |
